# Transcriptomics and Phenotypic Analysis of *gpr56* Knockout in Zebrafish

**DOI:** 10.3390/ijms24097740

**Published:** 2023-04-23

**Authors:** Luning Sun, Boyu Yang, Zheng Peng, Tianle Yang, Bin Qin, Jieyu Ao, Yanqun Yang, Jingling Wang, Lan Zheng, Huaping Xie

**Affiliations:** 1 Hunan International Joint Laboratory of Animal Intestinal Ecology and Health, Laboratory of Animal Nutrition and Human Health, College of Life Sciences, Hunan Normal University, Changsha 410081, China; 2 Hunan Provincial Key Laboratory of Animal Intestinal Function and Regulation, Changsha 410081, China; 3 Key Laboratory of Physical Fitness and Exercise Rehabilitation of Hunan Province, Hunan Normal University, Changsha 410081, China; 4 Heart Development Center, College of Life Science, Hunan Normal University, Changsha 410081, China

**Keywords:** *gpr56*, knockout, zebrafish, RNA-seq, differentially expressed genes, innate immunity, pancreas, motor ability

## Abstract

The adhesion G-protein-coupled receptor is a seven-transmembrane receptor protein with a complex structure. Impaired *GPR56* has been found to cause developmental damage to the human brain, resulting in intellectual disability and motor dysfunction. To date, studies on *gpr56* deficiency in zebrafish have been limited to the nervous system, and there have been no reports of its systemic effects on juvenile fish at developmental stages. In order to explore the function of *gpr56* in zebrafish, the CRISPR/Cas9 gene-editing system was used to construct a *gpr56*-knockout zebrafish. Subsequently, the differentially expressed genes (DEGs) at the transcriptional level between the 3 days post fertilization (dpf) homozygotes of the *gpr56* mutation and the wildtype zebrafish were analyzed via RNA-seq. The results of the clustering analysis, quantitative PCR (qPCR), and in situ hybridization demonstrated that the expression of innate immunity-related genes in the mutant was disordered, and multiple genes encoding digestive enzymes of the pancreatic exocrine glands were significantly downregulated in the mutant. Motor ability tests demonstrated that the *gpr56^−/−^* zebrafish were more active, and this change was more pronounced in the presence of cold and additional stimuli. In conclusion, our results revealed the effect of *gpr56* deletion on the gene expression of juvenile zebrafish and found that the *gpr56* mutant was extremely active, providing an important clue for studying the mechanism of *gpr56* in the development of juvenile zebrafish.

## 1. Introduction

G-protein-coupled receptors (GPCRs) are seven-transmembrane receptors that mediate signal transduction through the G protein and arrestins [1]. As the largest membrane protein family in the human genome, GPCRs have more than 800 members in humans [2,3]. As the second main type of GPCR, adhesion G-protein-coupled receptors (aGPCRs) have been studied; however, due to their complex structure, our understanding is still relatively insufficient [4]. Only three of the 33 currently identified aGPCRs have been found to contain a causative mutation, and GPR56 is one of them [5]. Moreover, GPR56 is the only aGPCR associated with brain malformations [6]. As a multifunctional aGPCR, GPR56 is involved in many physiological functions, such as brain development, myelination of the central nervous system, reproductive system development, the maintenance of hematopoietic stem cells, the formation of adipose tissue, the production and regulation of pancreatic/islet β cells, tumor growth and metastasis, and immune regulation [7,8,9].

It has been reported that *GPR56* mutation causes bilateral frontoparietal polymicrogyria (BFPP), a kind of cerebral cortex deformity [10]. Subsequently, cases of *GPR56* homozygous missense mutations, homozygous nonsense mutations, and compound heterozygous mutations have been reported with phenotypes of BFPP [11,12,13,14]. Patients with BFPP have cerebellar and pontine hypoplasia, which is clinically characterized by intellectual disability, seizures, motor developmental delay, and nonprogressive ataxia [15]. The measurement of movement behavior of *Gpr56^−/−^* mice showed that there was no obvious movement abnormality in the normal state, but the mutants showed movement disorder when they performed challenging movements [15].

In situ hybridization showed that the earliest expression of *gpr56* in zebrafish was around 11 h post fertilization (hpf) [16]. High-throughput real-time fluorescence quantitative PCR showed that *gpr56* was highly expressed in zebrafish embryos at 14 hpf and 24 hpf, expressed in juvenile fish at 3–21 dpf, and highly expressed in the intestine of adult fish [17]. At present, there are few studies on mutant zebrafish *gpr56*; the existing achievements have revealed that *gpr56* participates in the production of oligodendrocytes, myelin, and myelin sheath [4,18]. Zebrafish is one of the main model systems to study sports, and the changes in its movement speed and time can reflect abnormal development of the brain and nerves [19,20].

Using CRISPR/Cas9 gene-editing technology, we constructed three zebrafish lines with the *gpr56* gene knocked out. Transcriptome differences between homozygous *gpr56* mutation and wildtype juvenile fish were analyzed via high-throughput sequencing. We found that immune-related genes and some pancreas-specific genes of the *gpr56* mutant were abnormally expressed. A behavioral test found that *gpr56^−/−^* was too active.

## 2. Results

### 2.1. Construction of gpr56-Knockout Zebrafish Lines

**Figure 1:**
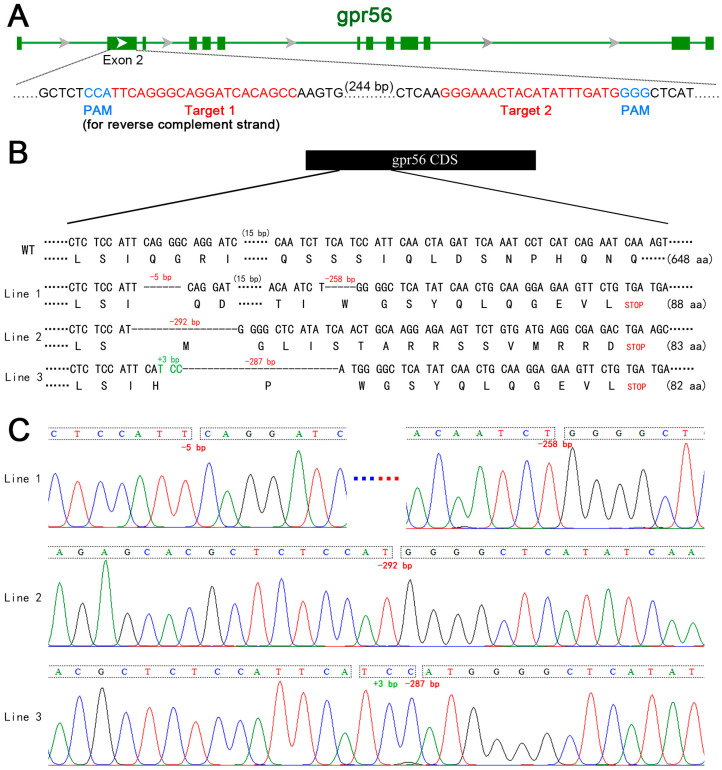
Schematic diagram of *gpr56* gene knockout in zebrafish. (**A**) Schematic diagram of sgRNA targeting used in *gpr56* gene knockout. The green horizontal line represents the genomic DNA of *gpr56*, the green rectangle represents the exons of *gpr56*, red shows the target sequence, and blue shows PAM [23]. (**B**) Schematic diagram of three heritable mutant alleles of *gpr56* generated by gene knockout and the protein sequences encoded by them. (**C**) Sequencing peaks of three mutant alleles of *gpr56*, in which A, G, C and T are shown by green, black, blue and red curves respectively.

To explore the role of the *gpr56* gene in the development of juvenile zebrafish, we constructed *gpr56*-knockout zebrafish lines. A pair of targeting sites of small guide RNAs (sgRNAs) located in exon 2 of gpr56 were designed, namely, Target 1 and Target 2 (Figure 1A). They were designed with the expectation that the CRISPR/Cas9 gene-editing system was used to introduce frameshift mutations into the coding sequence (CDS) of *gpr56* through nonhomologous end joining (NHEJ), resulting in translation errors and premature stop codons of *gpr56* [21,22]. A transcription template of the sgRNAs targeting exon2 of *gpr56* was synthesized via PCR (the primers used are shown in Table S1). Transcription was carried out in vitro using T7 polymerase, and then the product was purified. Cas9 protein and sgRNAs were co-injected into fresh, fertilized eggs of wildtype zebrafish. The chimeras in the surviving embryos were mated with wild zebrafish, and mutant alleles with the partial deletion of *gpr56* in the offspring were sequenced. Three zebrafish lines with the mutated alleles of the *gpr56* frameshift were identified via Sanger sequencing, and they were named Lines 1–3. Specifically, Line 1 contains two separate deletions (5 and 258 bp); a single deletion of 292 bp was found in Line 2; Line 3 included an insertion of 3 bp plus a deletion of 287 bp (Figure 1B,C). The heterozygotes of the three mutant lines were self-crossed to produce F2 homozygotes. As the mutant homozygotes of the above three knockout lines of *gpr56* did not show any obvious difference in appearance from that of the wildtype, the following experiments were all completed with Line 1.

### 2.2. Analysis of Transcriptome Sequencing Data

According to previous studies, both high-throughput quantitative real-time PCR and reverse-transcription PCR showed that *gpr56* was obviously expressed at 3 dpf or earlier [4,17]. Therefore, we performed transcriptome high-throughput sequencing on 3 dpf wild zebrafish juveniles and those with the homozygous *gpr56* mutation to explore the gene expression changes caused by the deletion of the *gpr56* gene.

**Figure 2:**
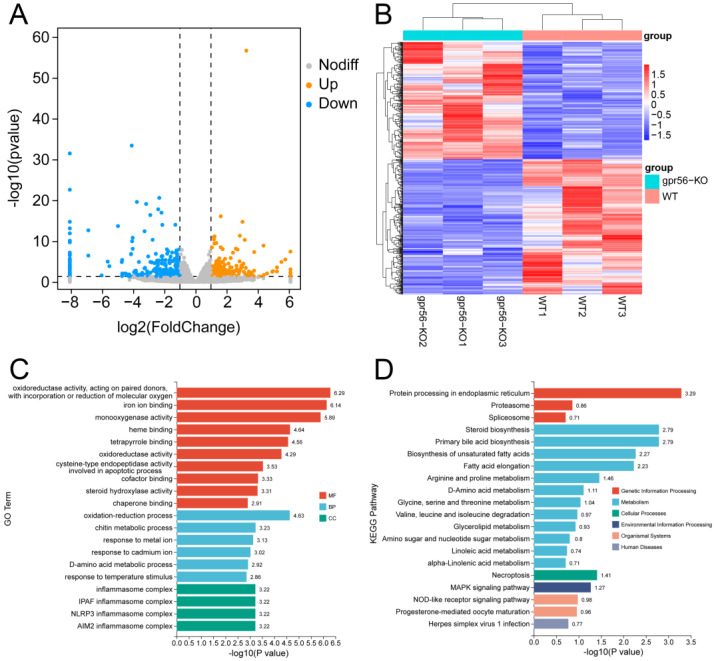
Effects of *gpr56* deletion on transcriptome of 3 dpf zebrafish. (**A**) Volcanic map of DEGs, in which two vertical, dotted lines in the figure distinguish DEGs that differed more than twofold, whereas DEGs above the horizontal, dotted line had a *p*-value < 0.05. (**B**) Heat map of DEGs, in which one row represents a gene and each column is a sample. Red represents high-expression genes, and blue represents low-expression genes. A darker color denotes a more significant difference. (**C**) GO enrichment analysis of DEGs, including molecular functions (MFs), biological processes (BPs), and cellular components (CCs). (**D**) Enrichment analysis of KEGG pathway of DEGs.

The results showed that, compared with the wildtype, 258 DEGs of the deletion homozygotes of *gpr56* were upregulated and 298 were downregulated, as displayed in the volcano map in Figure 2A. All DEGs are shown in the heat map in Figure 2B, in which gpr56-KO represents the homozygous *gpr56* knockout zebrafish. In order to further understand the effects of *gpr56* deletion on the biological functions of zebrafish, we carried out GO enrichment and KEGG pathway enrichment analysis on the DEGs. The most significant 20 GO items are shown in the histogram, including cofactor binding (GO: 0048037, MF), response to temperature stimulus (GO: 0009266, BP), and the inflammasome complex (GO: 0061702, CC) (Figure 2C). The top 20 KEGG pathways included steroid biosynthesis (DRE00100), glycerol lipid metabolism (DRE00561), and herpes simplex virus 1 infection (DRE05168) (Figure 2D).

The FPKM of gpr56 of 3 dpf *gpr56^−/−^* zebrafish was significantly downregulated compared to that of WT (Figure S4). Then, we used qPCR to analyze DEGs from the transcriptome of different batches of 3 dpf *gpr56^−/−^* and WT zebrafish. The results showed that *slc5a9*, *otod*, *gsdmeb*, *pigp*, *cthl*, and *bcam* all had the same difference trend as those in transcriptome sequencing, among which *slc5a9*, *pigp*, *cthl*, and *bcam* were significant (*p* < 0.05) (Figure S1).

### 2.3. Deletion of gpr56 Leads to an Imbalance of Innate Immunity-Related Gene Expression in Zebrafish Juveniles

**Figure 3:**
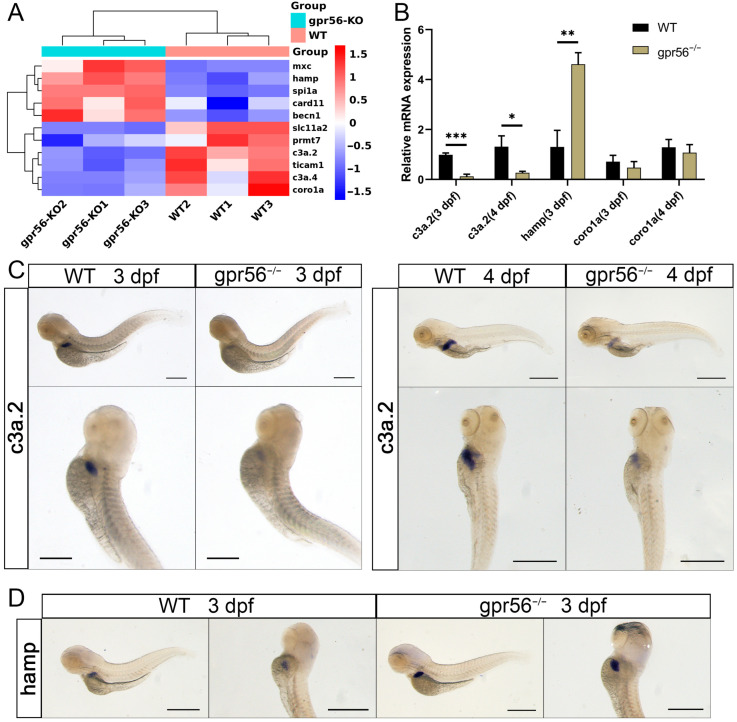
The deletion of *gpr56* affects the expression of innate immunity-related genes in zebrafish juveniles. (**A**) Cluster analysis of the expression of genes related to innate immunity. (**B**) qPCR relative quantification of the mRNA level of innate immune-related genes. Values plotted are means ± SD; * *p* < 0.05, ** *p* < 0.01, *** *p* < 0.001 (N = 3). (**C**) WISH representation of *c3a.2* in WT and *gpr56^−/−^* juveniles at 3 dpf and 4 dpf. (**D**) WISH representation of *hamp* in 3 dpf WT and *gpr56^−/−^* juveniles. Scale bar  =  500 μm.

In the further analysis of RNA-seq, we found that some genes related to innate immunity appeared in DEGs, and their changing trends were not consistent. Therefore, we selected some innate immune marker genes or related genes for cluster analysis (Figure 3A). The results of the qPCR showed that *c3a.2*, which encodes the complement component, was obviously downregulated in 3 dpf mutant zebrafish compared with WT, and this change continued in 4 dpf juvenile fish (Figure 3B). Contrary to *c3a.2*, *hamp*, which is used to encode hepcidin antibacterial peptide, was significantly upregulated in 3 dpf zebrafish (Figure 3B). Macrophage-labeled *coro1a* showed the same trend as RNA-seq in qPCR in 3 dpf and 4 dpf zebrafish, but there was no significant change (Figure 3B).

Next, we used WISH to detect the expression of *c3a.2* and *hamp* in juvenile fish. Compared with the wildtype, the signal intensity of *c3a.2* in 3 dpf and 4 dpf homozygous zebrafish with the *gpr56* mutation decreased obviously (Figure 3C), while that of *hamp* increased in 3 dpf zebrafish mutants (Figure 3D). It is worth noting that these two genes are specifically expressed in the liver. However, according to RNA-seq data, the expression of the liver marker gene fabp10a in the *gpr56* mutant did not seem to change significantly (Figure S2), which suggests that the development of the liver may not be significantly affected. To prove that the above results were not caused by CRISPR/Cas9 off-target effects, we also performed qPCR on 3 dpf juvenile fish of Line 2 with *gpr56* knocked out. As shown in Figure S3, the changing trends of *c3a.2* and *hamp* in mutant homozygotes of Line 2 compared with WT were similar to those of Line 1, suggesting that the changes in gene expression were specifically caused by the deletion of *gpr56*.

### 2.4. The Absence of gpr56 Causes Multiple Damages to Pancreatic Exocrine Secretion

**Figure 4:**
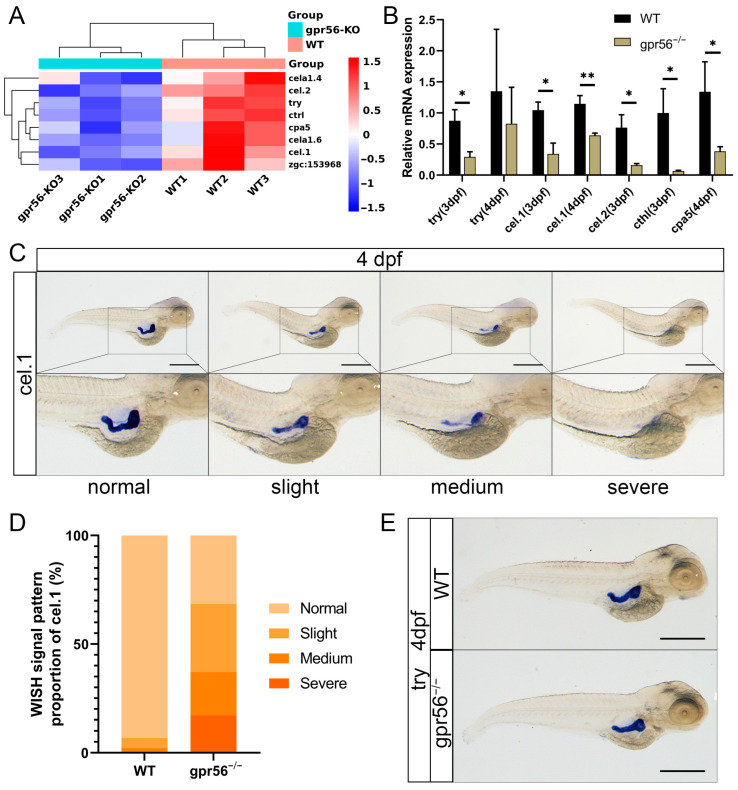
The deletion of *gpr56* affects the expression of pancreatic-specific genes in juvenile zebrafish. (**A**) Cluster analysis heatmap of pancreatic-specific expression genes. (**B**) qPCR detects the genes specifically expressed in pancreatic exocrine glands. Values plotted are means ± SD; * *p* < 0.05, ** *p* < 0.01 (N = 3). (**C**) Four WISH representative diagrams of *cel.1* in 4 dpf WT and *gpr56^−/−^* zebrafish: from normal to severe defects. (**D**) WT 4 dpf (n = 44) and *gpr56^−/−^* (n = 35) zebrafish, consistent with the above four *cel.1* expressions. (**E**) Representative WISH of *try* in 4 dpf WT and *gpr56^−/−^* zebrafish. Scale bar  =  500 μm.

Further analysis of the RNA-seq showed that many genes specifically expressed in the pancreas were obviously downregulated in the *gpr56* mutant, and most of them were located in the exocrine gland of the pancreas. Some genes expressed in the exocrine glands of the pancreas were clustered, including genes encoding components of trypsin, chymotrypsin, and carboxypeptidase [24,25] (Figure 4A). The qPCR analysis showed that *try* was significantly downregulated in 3 dpf *gpr56^−/−^* zebrafish compared with WT; however, at 4 dpf, the difference seemed to be restored (Figure 4B). In addition, *cel.1*, *cel.2*, and *cthl* were significantly downregulated in 3 dpf mutants, while *cel.1* and *cpa5* were significantly downregulated in 4 dpf mutants (Figure 4B). The 4 dpf juvenile zebrafish were determined to perform WISH to detect in situ expression patterns of pancreas-specific genes. When *cel.1* was detected by WISH, it was found that the expression of *cel.1* was downregulated to different degrees in different individuals of the 4 dpf mutant. Thus, the abnormal phenotype was divided into slight, medium, and severe defects (Figure 4C). Specifically, the normal phenotypic signal was plump and dark, appearing in most WT juvenile fish; the slight phenotypic signal was lighter than normal, with little loss; the medium phenotypic signal was characterized by massive loss and discontinuity; the severe phenotypic signal almost disappeared, or only maintained a small level. The normal expression of *cel.1* accounted for 31.43% of the total, slight defects accounted for 31.43%, medium phenotypes accounted for 20.00%, and severe phenotypes accounted for 17.14% (Figure 4D). In WT, the normal expression of *cel.1* accounted for 93.18%, mild defects accounted for 4.55%, moderate defects accounted for 2.27%, and there was no severe phenotype (Figure 4D). Furthermore, as a classic marker of differentiated pancreatic exocrine cells, *try* was also detected by WISH, and the results showed that it was not significantly reduced in 4 dpf *gpr56^−/−^* zebrafish (Figure 4E).

### 2.5. Deletion of gpr56 Makes 5 dpf Zebrafish More Active

**Figure 5:**
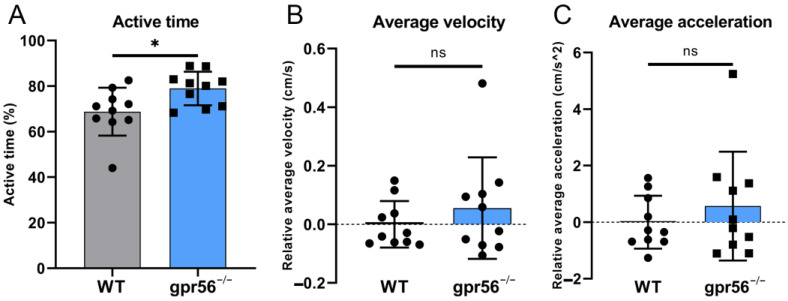
Motor ability test of 5 dpf WT and *gpr56^−/−^* zebrafish at 28 °C. (**A**) Activity time: the proportion of zebrafish activity time to the total test time. (**B**) Average speed, all data of which are zeroed to the average value of WT group. (**C**) Average acceleration, all data of which are zeroed to the average value of WT group. The circles record the test data and the values of the bars are their means ± SD; ^ns^ *p* > 0.05, * *p* < 0.05 (n = 10).

The original understanding of *gpr56* was that its mutations could lead to severe encephalopathy with concurrent motor dysfunction, which was later confirmed in mouse models [10,15]. Until now, there have been no reports on the motion of zebrafish with *gpr56* mutations. In order to investigate the effects of *gpr56* in zebrafish on movement in the early stage of development, we tested the motor ability of 5 dpf juvenile fish at 28 °C. Quantitative analysis of the motion data showed that 5 dpf *gpr56^−/−^* zebrafish had a higher proportion of moving time than WT zebrafish (Figure 5A), which means that the former was more active. However, there was no obvious difference in the average movement velocity (Figure 5B) and acceleration (Figure 5C) between mutant and WT zebrafish.

**Figure 6:**
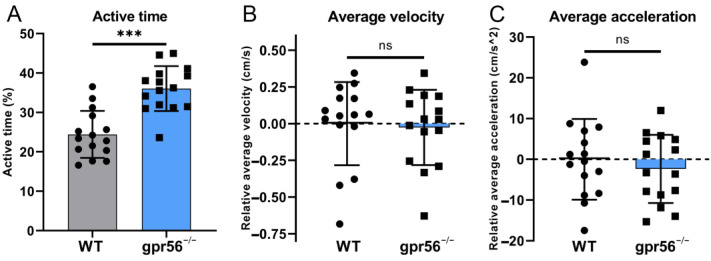
Motor ability test of 5 dpf WT and *gpr56^−/−^* zebrafish with additional tail stimulation at 23 °C. (**A**) Activity time: the proportion of zebrafish activity time to the total test time. (**B**) Average speed, all data of which are zeroed to the average value of WT group. (**C**) Average acceleration, all data of which are zeroed to the average value of WT group. The circles record the test data and the values of the bars are their means ± SD; ^ns^ *p* > 0.05, *** *p* < 0.001 (n = 15).

The above GO enrichment analysis shows that DEGs are enriched in “response to temperature stimulus” (Figure 2C), suggesting that the *gpr56* mutant zebrafish may exhibit more severe abnormalities than WT zebrafish at inappropriate temperatures. Thus, 5 dpf *gpr56^−/−^* and WT zebrafish were placed in 23 °C conditions for 1 h and maintained at this temperature for the motor ability test. Due to the low motor frequency of the juvenile fish under this condition, 30 s before each fish was tested, it was given a uniform tail-touching stimulus to increase its activity. Test results showed that this condition exacerbated the difference in activity between *gpr56^−/−^* and WT zebrafish, even though their activity time ratio was lowered (Figure 6A). Nevertheless, there is still no significant difference in the average velocity (Figure 6B) and acceleration (Figure 6C) between the two groups.

## 3. Discussion

In this study, taking zebrafish as a model animal, we knocked out *gpr56* and reported for the first time that the whole-body RNA-seq of mutant homozygous juvenile fish was used to detect abnormal gene expression at the transcriptional level.

The zebrafish lines with *gpr56* knockout we constructed were with open reading frame (ORF) frameshifts of *gpr56*, which led to Gpr56 protein not being normally translated. Theoretically, the mRNA of the truncated *gpr56* in the mutant lines would be transcribed, but the original intact Gpr56 protein no longer existed. As *gpr56^−/−^* zebrafish do not show obvious abnormalities across the life cycle, it is difficult to carry out research. According to previous studies, we determined that *gpr56* was clearly expressed at 3 dpf; thus, we hoped to find more research points through RNA-seq at 3 dpf. The significant reduction in the FPKM of gpr56 in the juvenile of *gpr56^−/−^* should be due to accelerated degradation of mRNA containing premature termination codons (PTCs), a mechanism known as non-sense-mediated mRNA decay (NMD) [26,27]. We found that there are many genes related to innate immunity in DEGs. The innate immune system is the first line of defense against infection or tissue damage, and its related cells and molecules are rapidly activated when encountering microorganisms or other “danger signals”, which is crucial to maintaining a healthy tissue microenvironment [28]. The innate immune system has various components, with soluble recognition molecules (including natural antibodies, pentapeptides, and the complement system), as well as cellular components (including phagocytes such as macrophages, innate lymphocytes, antigen-presenting cells, and killer cells) [28,29]. In fact, it is not surprising that the absence of *gpr56* changes the expression of innate immunity-related genes. It has been demonstrated that *GPR56* is expressed in T cells and NK cells, is an important regulator of their cellular development, migration, cytotoxic, and antiviral properties, and thus, participates in the coordination of innate and acquired immune responses [30,31,32,33]. Our study complemented the effects of *gpr56* deletions on innate immunity in vertebrates from a gene expression perspective. Macrophages are a ubiquitous cellular component and exist in all tissues and body parts under steady-state physiological conditions, playing a decisive role in the response of intracellular bacteria [34,35]. *Coro1A* is abundant on macrophage phagocytes [36], which are markers of macrophages. *CARD11* mediates the activation of NF-κB induced by antigen receptors to participate in innate and adaptive immunity [37,38]. *Prmt7* is a type III arginine methyltransferase that negatively regulates MAVS-mediated antiviral signal transduction to participate in innate immunity [39,40]. *BECN1*, as a key regulator of autophagy, is a part of the innate immune response [41,42,43,44]. The complement system is a powerful collection of over 40 serine proteases, receptors, and modulators that amplify dangerous signals and enhance inflammatory responses [45]. Complement C3 is the confluence point in the complement regulatory network that promotes the amplification of complement response, plays a direct effect function, and helps coordinate the downstream immune response [46]. *Hamp* encodes hepcidin, an antibacterial peptide that is mainly produced by the liver and released into the serum [47,48]. Hepcidin, which functions as both an antibacterial peptide and a hormone modulator of iron homeostasis, can induce a reduction in circulating iron, ultimately limiting the availability of iron to invading microorganisms and, thus, contributing to host defense [49]. It is worth noting that *c3a.2* and *hamp*, two important components of innate immunity that are enriched in liver specificity, actually have opposite differential expression. That is, *c3a.2* is downregulated while *hamp* is upregulated. However, both are positive modulators that contribute to the innate immune response, which are now regulated in the opposite direction, possibly as a result of one being affected by the absence of *gpr56* and the other being decompensated for by a complex cascade.

In fact, it is not surprising that the deletion of zebrafish *gpr56* affects its pancreatic exocrine gland, because it has been confirmed that mouse *Gpr56* is highly expressed in pancreas, and human *GPR56* is the most abundant GPCR in isolated human islets. As the earliest expressed marker of pancreatic exocrine glands, *try* first appeared in the 48 hpf in cells around the single islet [50,51]. In the *gpr56* mutant, expression of *try* and several other genes representative of pancreatic exocrine glands was significantly reduced at 3 dpf, indicating impaired development of pancreatic exocrine glands. When at 4 dpf, the low expression of mutant *try* was restored while *cel.1* and *cpa5* maintained low expression. This suggests that the direct causes for the downregulation of the expression of various pancreatic exocrine gland components may be different because their downregulation is not synchronous. cpa5 is a marker of pancreatic acinar cells and pancreatic exocrine that begins to be expressed in 60 hpf juvenile [25]. The human *CEL* gene encodes carboxyester lipase, which is a lipolytic enzyme that can hydrolyze cholesterol esters, triacyls, diacyls, monoacylglycerols, phospholipids, lysophospholipids, and ceramides [52]. Zebrafish *cel.1* and *cel.2*, as orthologs of *CEL* genes, are generally expressed in exocrine glands after 3 dpf, and some of their functions are highly conserved [53]. Knocking down *cel.1* and *cel.2* leads to the obstruction of embryo development, yolk sac retention, a reduction in free fatty acids, brain development damage, and even deformity [53]. We speculated that the downregulation of *CEL* might be a potential factor for brain dysplasia in *GPR56* mutant mammals, provided that lower expression of *CEL* was also present in *GPR56* mutant mammals. The expression patterns of early pancreatic markers, including ins and pdx1, showed that two cell groups converged at the midline between the 14- and 18-somite stages of zebrafish, and formed a single pancreatic tissue bud at the fourth segment [50]. Among them, pdx1 was first detected at the 10-somite stage, which means that the development of zebrafish pancreas may begin at around 14 hpf at the earliest [50]. This is very close to the time when the expression of gpr56 was first detected (11 hpf), indicating that the abnormality of the pancreas of *gpr56^−/−^* zebrafish may have appeared as early as its initial development. The specific mechanism of *gpr56^−/−^* zebrafish pancreatic exocrine gland defect will be the focus of our next study.

In the non-difficult athletic ability test, unlike humans, the absence of *gpr56* does not have a negative impact on the motor ability of zebrafish, but makes them more active. There is a possibility that the *gpr56^−/−^* zebrafish will show dyskinesia when performing challenging and difficult sports, just like the mouse model with *gpr56* mutation [15]. In future studies, a set of challenging motion detection schemes for juvenile zebrafish can be designed to explore the potential motor problems of *gpr56^−/−^* zebrafish.

In conclusion, we assessed the effect of *gpr56* gene deletion on systemic gene expression in juvenile zebrafish using quantitative techniques such as RNA-seq, qPCR, and WISH, and found that the juvenile zebrafish mutant *gpr56* had the characteristics of congenital immune-related gene expression disorder, inhibited development of pancreatic exocrine glands, and more activity. This study provided a novel perspective for exploring the role of gpr56 in the development of juvenile zebrafish.

Future studies should reveal the molecular mechanisms of the downregulation of many digestive enzyme components in the pancreatic exocrine glands and the causes of innate immune disorders, as well as identify the internal mechanism that causes the *gpr56* mutant zebrafish to become more active.

## 4. Materials and Methods

### 4.1. Experimental Zebrafish and Gene Knockout

The Tu strain of zebrafish was maintained, reared, and propagated at 28.5 °C, as previously described [54]. The breeding of zebrafish is carried out by moving one female fish and one male fish into a breeding vessel and separating them overnight with a transparent plastic partition. The plastic partition was removed at 9:00 a.m. the next day to allow zebrafish to mate for 1 h, and then the embryos were collected.

The zebrafish *gpr56* knockout was performed using the CRISPR/Cas9 gene-editing system. Specifically, PCR was performed using the sgRNA template (Table S1) (ssDNA) as a template to synthesize the transcription templates of the sgRNAs. The reverse primer used for the reaction was sgRNA-primer-R (Table S1), and the two forward primers containing the T7 promoter and target sequence were sgRNA-primer-F1 and sgRNA-primer-F2 (Table S1), respectively. Two sgRNAs were synthesized by in vitro transcription with a Maxiscript T7 transcription kit (AM1314, Thermo Fisher Scientific, Waltham, MA, USA). sgRNAs (each with a final concentration of 40 ng/μL) were co-injected with Invitrogen TrueCut Cas9 v2 (A36499, Thermo Fisher Scientific (final concentration of 300 ng/μL)) into the fertilized egg at the single-cell stage. Every zebrafish with a *gpr56* knockout strain involved in this study underwent PCR using forward primer *gpr56*-F and reverse primer *gpr56*-R, as shown in Table S1, to identify its genotype. This step occurs before hybridization or any animal experiment.

### 4.2. RNA-seq Transcriptome Analysis

Zebrafish at 3 dpf were collected, and 80 fish were stored as an independent biological replicate at −80 °C. Each group in RNA-seq was derived from a single biological replicate consisting of a collective of 80 fish. Then, RNA-seq and data analysis were performed at Personalbio (Shanghai Personal Biotechnology, Co. Ltd. Shanghai, China) as described previously [55]. The DEGs were analyzed by DESeq [56], and the conditions for screening the DEGs were as follows: expression difference multiple |log2 fold change| > 1, and significance *p*-value < 0.05.

The volcanic map of the DEGs was drawn with the ggplots2 software package (version 2.2.0) in R language [57]. The clustering analysis of DEGs was carried out using the Pheatmap software package (version 1.0.8) of R language. We used topGO (versioin 2.40.0) for GO enrichment analysis [58]. During the analysis, the gene list and gene number of each term were calculated using the differential genes annotated by GO term, and then the *p*-value was calculated using the hypergeometric distribution method (the criterion for significant enrichment was *p*-value < 0.05). We used clusterprofiler (version 3.16.1) for the KEGG enrichment analysis [59]. During the analysis, the gene list and gene number of each pathway were calculated using the differential genes annotated by the KEGG pathway, and then the *p*-value was calculated by the hypergeometric distribution method (the criterion for significant enrichment was a *p*-value <0.05).

### 4.3. qPCR Detection

The expression of related genes was detected by real-time fluorescent qPCR. Each group contained three replicates, with 20 zebrafish per replicate. Total RNA was extracted using the MiniBest Universal RNA ExtractionKit (9767, Takara, Dalian, China). It was reverse-transcribed to cDNA using the PrimeScript RT reagent kit (RR047A, Takara). The expression of these genes was also regulated using two internal controls (*actb1* and *eef1a1l1* genes). The qPCR was performed using the QuantStudio 5 Real Time PCR system (Thermo Fisher Scientific), and data were analyzed using the QuantStudio Design and Analysis software (version 1.5.2). The primers of the target genes including *c3a.2*, *hamp*, *coro1a*, *cel.1*, *try*, *cel.2*, *cthl*, *cpa5*, *slc5a9*, *otoa*, *gsdmeb*, *pigp* and *bcam* are shown in Table S2. The relative gene expression values were calculated using the ΔΔCT method [60].

### 4.4. Synthesis of RNA Probes and Whole In Situ Hybridization (WISH)

To prepare an mRNA antisense probe, a portion of the mRNA sequence of the gene was amplified using PCR. The forward primers and the reverse primers with T7 promoter sequences of *c3a.2*, *hamp*, *cel.1*, and *try* are shown in Table S3. Digoxin-labeled antisense RNA probes were synthesized using the purified PCR products as transcription templates. Zebrafish juvenile developed to specific stages were fixed overnight in 4% paraformaldehyde and stored in 100% methanol. As previously described, the location and level of mRNA expression were detected by WISH [61].

### 4.5. Imaging and Image Analysis

The WISH-stained juveniles were imaged using a fluoroscopic microscope (Leica M205FA, Wetzlar, Germany). All the images were taken using the Leica Application Kit imaging software (version 3.2.0).

### 4.6. Motor Ability Test

An exercise video of the juveniles was taken using an industrial camera (IDS, Obersulm, Germany) for approximately 100 s, and the motor ability analysis was performed using the lolitrack software (version 4, Loligo, Tjele, Denmark). Additional tail stimulation was performed by gently touching the tail of the juvenile zebrafish 10 times laterally with a small tip. After the tail stimulation was applied, the juveniles were allowed to stand for 30 s before the exercise video was taken. All the single data of average velocity and average acceleration were zeroed into the average value of the WT control group; that is, the average value of the WT group was subtracted from each datum. The relative average velocity and acceleration were calculated in this way.

### 4.7. Statistical Analysis

The significance of the data of qPCR and motor ability test was tested by unpaired t-test (^ns^ *p* > 0.05, * *p* < 0.05, ** *p* < 0.01, *** *p* < 0.001).

## Data Availability

The data presented in this study are available in the article and the Supplementary Materials. The RNA-seq data of this study are deposited in the NCBI Sequence Read Archive (SRA) with the BioProject accession number PRJNA906198.

## Supplementary Materials

The following supporting information can be downloaded at https://www.mdpi.com/article/10.3390/ijms24097740/s1.
