# Supplementary material for: Transcriptomics and Phenotypic Analysis of gpr56 Knockout in Zebrafish
Source: Int J Mol Sci. 2023 Apr 23;24(9):7740. doi: 10.3390/ijms24097740 (PMC10178538; doi:10.3390/ijms24097740)
Supplement: Supplementary file 1 [file ijms-24-07740-s001.zip › ijms-2343182-supplementary.pdf]

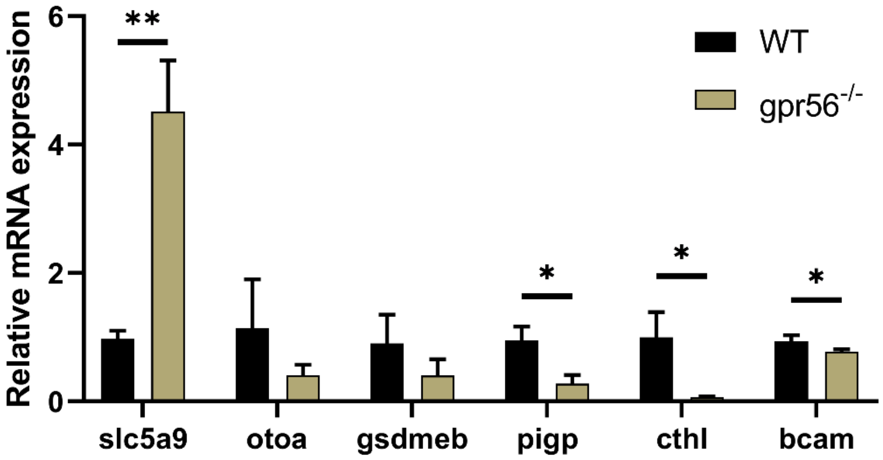

Figure S1: qPCR quantified the relative mRNA expression level of the selected genes. Values plotted are means ± SD, *$p < 0.05$，**$p < 0.01$ (N=3).

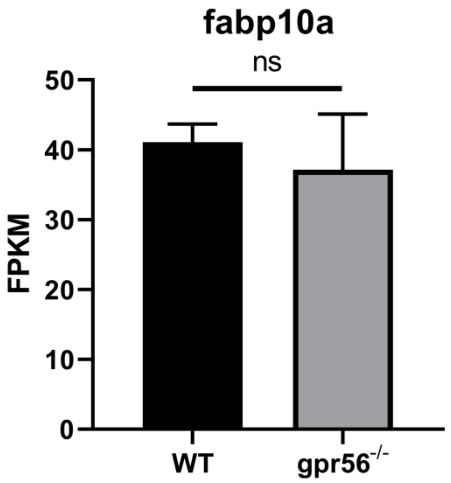

Figure S2: The FPKM of fabp10a obtained by RNA-seq data analysis. Values plotted are means ± SD, $^{ns}p > 0.05$ (N=3).

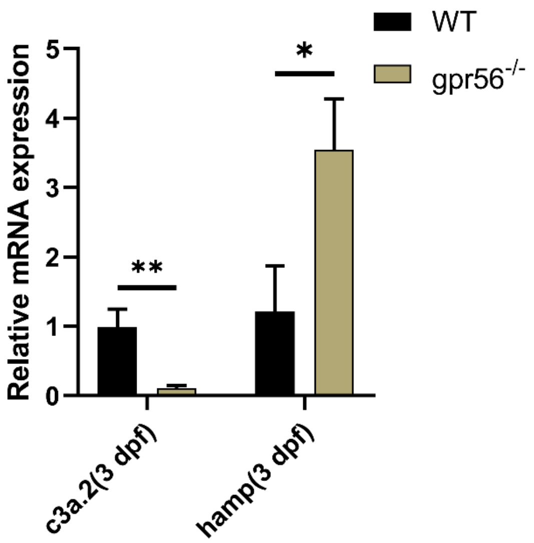

Figure S3: qPCR quantified the relative mRNA expression level of c3a.2 and hamp in Line 2. Values plotted are means ± SD, *p < 0.05， **p < 0.01 (N=3).

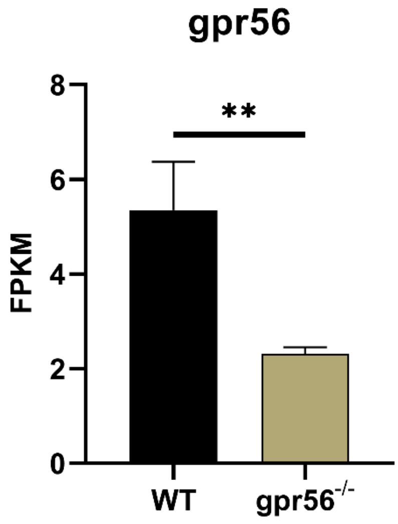

Figure S4: The FPKM of gpr56 obtained by RNA-seq data analysis. Values plotted are means ± SD, **p > 0.01 (N=3).

Table S1: primers used for gene knockout and their sequences.

Primer	Sequences (5′→ 3′)
sgRNA-Template	TTTTAGAGCTAGAAATAGCAAGTTAAAATAAGGCTAGTCCGTTATCA ACTTGAAAAAGTGGCACCGAGTCGGTGCTTT
sgRNA-primer-F1	GCGTAATACGACTCACTATAGGCTGTGATCCTGCCCTGAAGTTTTAGA GCTAGAAATAG
sgRNA-primer-F2	GCGTAATACGACTCACTATAGGGAAACTACATATTTGATGGTTTTAG

	AGCTAGAAATAG
sgRNA-primer-R	AAGCACCGACTCGGTGCCACT
gpr56-F	AGATGTGTGGAAAGTGGCTCC
gpr56-R	CAGTCTCGCCTCATCACAGA

Table S2: primers used for qPCR and their sequences.

Primer	Sequences (5′→ 3′)
c3a.2-qPCR-F	ACGCAATAAAGGCAGAGAAAGG
c3a.2-qPCR-R	AAAAGTCGGCAGCACGTATTCT
hamp-qPCR-F	ACAGCCGTTCCCTTCATACAG
hamp-qPCR-R	CAGCCTTTATTGCGACAGCA
coro1a-qPCR-F	TGGAACAGAAATGGCTCGCA
coro1a-qPCR-R	GCTCACCGAAGTTGTTGGGAT
cel.1-qPCR-F	CTGTCTGATGATGATGGCGA
cel.1-qPCR-R	TGTTTTCAGGACACCTTCCCAG
try-qPCR-F	CTTCTGGCTCTTTTCGCTGTG
try-qPCR-R	ACCTGGACACGGGACTTG
cel.2-qPCR-F	GGGAAAGTGAGGCTTGCTAC
cel.2-qPCR-R	TGGACCTCCTCTTCAGTTGT
cthl-qPCR-F	GAGGCTGTTCTGGGATGGTC
cthl-qPCR-R	ATGAGGCATAGTCGCTGGAAG
cpa5-qPCR-F	CTACACCCACACCAAAGACCG
cpa5-qPCR-R	CCTCCAAAACCAGCATCCCA
slc5a9-qPCR-F	TGGCAGTTGGAATATGGTCTT
slc5a9-qPCR-R	TGGCAGTTGGAATATGGTCTT
otoa-qPCR-F	CACTGCTGAATGCGTTTCGT
otoa-qPCR-R	TCCCTAATTCGCCAAGGTTGA
gsdmeb-qPCR-F	CGAGGTGCCTGAACTGAAGG
gsdmeb-qPCR-R	TGCTGGCTTACTCATCTCGTCT
pigp-qPCR-F	CGCAGAAACACAAGGAAGCA
pigp-qPCR-R	GACCACATACAGCAAGAAGCC
bcam-qPCR-F	TTCTGGCAAAGAGTCGGTGA
bcam-qPCR-R	TCAGAATTGGCAGGTGTGGG

Table S3: primers used for WISH and their sequences.

Primer	Sequences (5′→ 3′)
c3a.2-WISH-F	GAAAGTGGTCACGCGGTACA
c3a.2-WISH-T7R	GCGTAATACGACTCACTATAGGGGTCATTGTTTCACTGCCTGC
hamp-WISH-F	CAGAGCCGAGCAGAAGAAAAGT
hamp-WISH-T7R	GCGTAATACGACTCACTATAGGGAGCCTGCATTTATACCCGTTTTC
cel.1-WISH-F	TCTTGCTACTGATGTTATCGGC

cel.1-WISH-T7R	GCGTAATACGACTCACTATAGGGATGTGTGCGTCCATGTCGTT
try-WISH-F	CACACCACTCAACAGCTTCAC
try-WISH-T7R	GCGTAATACGACTCACTATAGGGGTAAACACCGGGCTTGTTCC
